# Evaluation of the Effectiveness of Surveillance and Containment Measures for the First 100 Patients with COVID-19 in Singapore — January 2–February 29, 2020

**DOI:** 10.15585/mmwr.mm6911e1

**Published:** 2020-03-20

**Authors:** Yixiang Ng, Zongbin Li, Yi Xian Chua, Wei Liang Chaw, Zheng Zhao, Benjamin Er, Rachael Pung, Calvin J. Chiew, David C. Lye, Derrick Heng, Vernon J. Lee

**Affiliations:** ^1^Ministry of Health, Singapore; ^2^National Centre for Infectious Diseases, Singapore; ^3^Tan Tock Seng Hospital, Singapore; ^4^Lee Kong Chian School of Medicine, Singapore; ^5^Yong Loo Lin School of Medicine, Singapore; ^6^Saw Swee Hock School of Public Health, Singapore.

*On March 13, 2020, this report was posted online as an *MMWR *Early Release.*

Coronavirus disease 2019 (COVID-19) was first reported in Wuhan, China, in December 2019, and has since spread globally, resulting in >95,000 confirmed COVID-19 cases worldwide by March 5, 2020 (*1*). Singapore adopted a multipronged surveillance strategy that included applying the case definition at medical consults, tracing contacts of patients with laboratory-confirmed COVID-19, enhancing surveillance among different patient groups (all patients with pneumonia, hospitalized patients in intensive care units [ICUs] with possible infectious diseases, primary care patients with influenza-like illness, and deaths from possible infectious etiologies), and allowing clinician discretion (i.e., option to order a test based on clinical suspicion, even if the case definition was not met) to identify COVID-19 patients. Containment measures, including patient isolation and quarantine, active monitoring of contacts, border controls, and community education and precautions, were performed to minimize disease spread. As of March 5, 2020, a total of 117 COVID-19 cases had been identified in Singapore. This report analyzes the first 100 COVID-19 patients in Singapore to determine the effectiveness of the surveillance and containment measures. COVID-19 patients were classified by the primary means by which they were detected. Application of the case definition and contact tracing identified 73 patients, 16 were detected by enhanced surveillance, and 11 were identified by laboratory testing based on providers’ clinical discretion. Effectiveness of these measures was assessed by calculating the 7-day moving average of the interval from symptom onset to isolation in hospital or quarantine, which indicated significant decreasing trends for both local and imported COVID-19 cases. Rapid identification and isolation of cases, quarantine of close contacts, and active monitoring of other contacts have been effective in suppressing expansion of the outbreak and have implications for other countries experiencing outbreaks.

On January 2, 2020, days after the first report of the disease from China, the ministry of health (MOH) in Singapore, a small island city-state in Southeast Asia with a population of approximately 5.7 million, developed a local case definition (Supplementary Table, https://stacks.cdc.gov/view/cdc/85735) and advised all medical practitioners to be vigilant for suspected COVID-19 patients (*2*). A confirmed case was defined as a positive test for SARS-CoV-2, the virus that causes COVID-19, by reverse transcription–polymerase chain reaction (RT-PCR) (*3*), or a positive viral microneutralization antibody test using a SARS-CoV-2 virus isolate (BetaCoV/Singapore/2/2020; GISAID accession 76 number EPI_ISL_407987) and conducted using previously published protocols (*4*). At hospitals, patients with suspected COVID-19 received chest radiographs and RT-PCR testing on at least two nasopharyngeal swabs collected 24 hours apart (*5*). Physicians are mandated to report all suspected and confirmed COVID-19 patients through a centralized disease notification system.

The case definition was updated five times following the outbreak’s start to adapt to the evolving global situation (Supplementary Table, https://stacks.cdc.gov/view/cdc/85735). The MOH carried out contact tracing around confirmed cases to identify persons who might have been infected. Contacts with fever (temperature ≥100.4°F [≥38°C]) or respiratory symptoms were transferred directly to a hospital for further evaluation and testing. Close contacts were defined as having close (within 6.6 ft [2 m]) and prolonged (generally ≥30 minutes) contact with the COVID-19 patient. Contacts at lower risk were persons who had some interactions with the COVID-19 patient for shorter periods of time. Asymptomatic close contacts were placed under compulsory quarantine for 14 days, and contacts at lower risk were placed under active monitoring. All contacts were assessed by telephone for fever or respiratory symptoms by public health officials during the quarantine or monitoring period, thrice daily for close contacts and once daily for contacts at lower risk. Contacts who became symptomatic were transferred to a hospital. Surveillance was enhanced in late January 2020 by testing the following groups for COVID-19: 1) all hospitalized patients with pneumonia (later expanded to include patients with pneumonia evaluated in primary care settings); 2) ICU patients with possible infectious causes as determined by the physician; 3) patients with influenza-like illness at sentinel government and private primary care clinics included in the routine influenza surveillance network; and 4) deaths from possible infectious causes. In addition, medical practitioners could choose to test patients if there was clinical (e.g., prolonged respiratory illness with unknown cause) or epidemiologic (e.g., association with known clusters) suspicion.

The effectiveness of Singapore’s surveillance and containment efforts was assessed from the outbreak’s start until February 29 by calculating the 7-day moving average of the interval from symptom onset to isolation in hospital or quarantine. This measure provides an indication of the time spent within the community when a person with COVID-19 is potentially infectious. Differences in the percentages of cases detected through the different surveillance components were tested using the chi-squared or Fisher’s exact test. All analyses were conducted using R statistical software (version 3.5.1; The R Foundation).

Among the first 100 confirmed COVID-19 cases in Singapore, the average patient age was 42.5 years (median = 41 years; interquartile range [IQR] = 34–54 years) (Table). The majority (72%) of patients were aged 30–59 years, and 60% of patients were male. RT-PCR confirmed 99% of cases, and one case was confirmed by viral microneutralization testing. Twenty-four cases were imported, and the rest resulted from local transmission. Fifteen patients were ever in the ICU; no deaths have been reported to date. Contact tracing contributed to the primary detection of approximately half (53%) of COVID-19 patients. Another 20 (20%) patients were identified at general practitioner clinics or hospitals because they met the case definition; 16 were identified through enhanced surveillance (15 from pneumonia surveillance and one from the ICU), and another 11 through medical providers’ clinical discretion. No patients were identified through surveillance for influenza-like illness. A significant difference was found in the percentage of cases detected by the various surveillance methods, depending on whether the cases were linked to another COVID-19 patient or by travel to China, compared with cases that could not be linked to another case (p<0.001). Among linked cases, the largest proportion (62.7%) was detected through contact tracing, whereas among unlinked cases, the largest proportion of cases (58.8%) was detected through enhanced surveillance (Table). The earliest symptom onset date reported by a COVID-19 patient was January 14 (Figure 1). The epidemic curve peaked on January 30, when nine patients were identified, before declining to two to five patients per day on February 11 and continuing forward. International importations accounted for a majority of cases at the outbreak’s start before more local cases were detected. The mean interval from symptom onset to hospital isolation or quarantine was 5.6 days (median = 5 days; IQR = 2–8 days). The 7-day moving average of the interval from symptom onset to isolation declined significantly across the study period for both imported and local cases, from 9.0 and 18.0 days to 0.9 and 3.1 days, respectively (p<0.001) (Figure 2). Among the 53 patients first identified through contact tracing, 13 (24.5%) were contacted on or before the date of symptom onset.

**TABLE:** Characteristics of coronavirus disease 2019 (COVID-19) cases, by linkage to other known cases (N = 100) — Singapore, January–February 2020

Characteristic	No. (%) of COVID-19 cases			P-value
	Total	Linked*	Unlinked^†^	
**Age group (yrs)**				
<30	**17 (17.0)**	17 (20.5)	0 (—)	0.12
30–39	**28 (28.0)**	23 (27.7)	5 (29.4)	
40–49	**20 (20.0)**	16 (19.3)	4 (23.5)	
50–59	**24 (24.0)**	20 (24.1)	4 (23.5)	
≥60	**11 (11.0)**	7 (8.4)	4 (23.5)	
**Sex**				
Male	**60 (60.0)**	46 (55.4)	14 (82.4)	0.06
Female	**40 (40.0)**	37 (44.6)	3 (17.6)	
**Ethnic group**				
Chinese	**87 (87.0)**	74 (89.2)	13 (76.5)	0.21
Indian	**6 (6.0)**	4 (4.8)	2 (11.8)	
Malay	**2 (2.0)**	1 (1.2)	1 (5.9)	
Other	**5 (5.0)**	4 (4.8)	1 (5.9)	
**Primary detection method**				
Contact tracing	**53 (53.0)**	52 (62.7)	1 (5.9)	<0.001
Case definition at medical consult	**20 (20.0)**	16 (19.3)	4 (23.5)	
Enhanced surveillance	**16 (16.0)**	6 (7.2)	10 (58.8)	
Provider clinical discretion	**11 (11.0)**	9 (10.8)	2 (11.8)	

* Patients who were epidemiologically linked to other COVID-19 patients or had recent travel to China.

^†^ Patients whose source of infection could not be determined.

**FIGURE 1:**
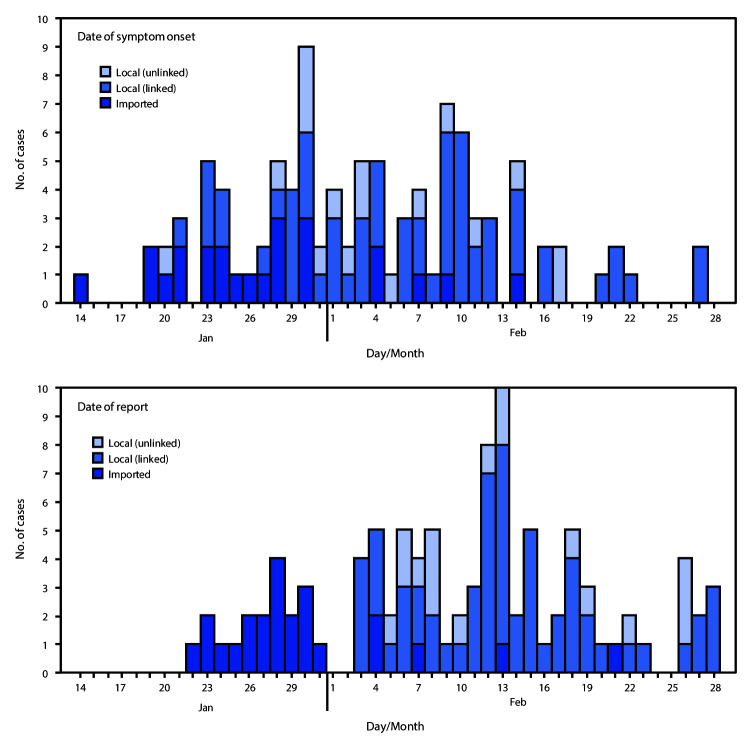
Date of symptom onset and date of report for cases of coronavirus disease 2019 (COVID-19) (N = 100), based on importation and linkage*^,†^ status — Singapore, January 14–February 28, 2020 * Linked patients defined as those who were found to be epidemiologically linked to other COVID-19 patients or who had recent travel to China. ^†^ Unlinked patients defined as those whose source of infection could not be determined.

**FIGURE 2:**
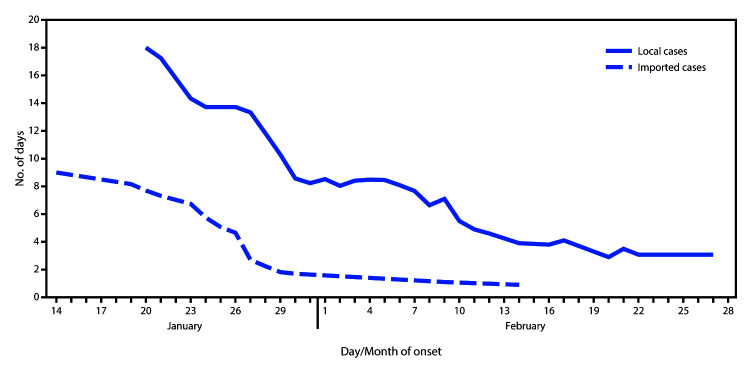
Interval from symptom onset to isolation or hospitalization (7-day moving average), of coronavirus disease 2019 (COVID-19 cases) (N = 100), by importation status — Singapore, January 14–February 28, 2020

## Discussion

In this assessment of the measures that Singapore, a small city-state, put in place to identify COVID-19 patients and contain disease spread in the early outbreak phase, approximately one quarter of cases were detected through enhanced surveillance among hospitalized patients with pneumonia and ICU patients (16 cases [16%]) and through providers’ clinical discretion (11 [11%]). A recent study considered Singapore to have the highest surveillance capacity for COVID-19 among all countries (*6*). The study estimated that if other countries had similar detection capacities as Singapore, the global number of imported cases detected would be 2.8 times higher than the observed current number. The surveillance methods in Singapore complemented one another to identify infected persons, with the overlapping components constituting safety nets; none of the methods alone would have detected all patients. The case definition was important for clinicians to use as a foundation, and active case finding around COVID-19 patients through contact tracing was useful in detecting new patients early for isolation.

The enhanced surveillance measures of SARS-CoV-2 testing of all patients with pneumonia, surveillance of ICU patients with severe illness and deaths potentially attributable to COVID-19, and clinical discretion in requesting testing were all important in detecting initially unlinked patients for further investigation. Adoption of multiple surveillance mechanisms can ensure broad coverage because each missed case can lead to chains of transmission that might be difficult to contain subsequently.

Singapore has implemented aggressive measures to contain local transmission of COVID-19. After an initial increase in locally transmitted cases, the number of newly identified cases decreased after approximately 1 month, determined by symptom onset dates. This decrease is likely a result of the early implementation of surveillance and detection measures while the numbers of patients were still small and individual-level containment was possible; a larger number of cases would have driven community transmission. The decline in the 7-day moving average of interval from onset to isolation in hospital and quarantine was also indicative of efforts to contain disease spread across time.

Singapore has also implemented other measures to reduce the spread of COVID-19. To prevent imported cases from seeding local transmission, border control measures included temperature screening initially for travelers on flights from Wuhan before expanding to include all travelers entering Singapore at air, sea, and land checkpoints (*7*). Short-term visitors with travel in the past 14 days to selected countries or regions (initially mainland China and later expanded to South Korea, northern Italy, and Iran) were denied entry; Singapore residents returning from these areas were placed under a mandatory 14-day self-quarantine. To reduce community spread, public education messages were focused on personal hygiene and seeking early medical care and self-isolation when having respiratory symptoms. As of March 5, 2020, schools have not closed because there was no widespread community transmission in Singapore and few cases among children; precautionary measures such as reducing mixing across classes or schools have been implemented to limit possible disease transmission.

The findings in this report are subject to at least three limitations. First, the 7-day moving average interval from symptom onset to isolation could fluctuate for recent dates as additional patients are detected and might be insufficient as a single measure to evaluate the effectiveness of containment. Further indicators to assess effectiveness of containment measures should be investigated. Nevertheless, the downward trend was significant from the outbreak’s start until early February. Second, the case detection methods were primarily focused on symptomatic patients. Further studies are needed to assess the number of asymptomatic patients in the community and their potential to transmit disease and whether additional measures targeting asymptomatic patients would have resulted in further case reductions. Finally, generalizability of results is limited because of the small sample size and lack of cases in settings such as long-term nursing facilities and health care settings.

Singapore implemented strong surveillance and containment measures, which appear to have slowed the growth of the outbreak. These measures might be useful for detection and containment of COVID-19 in other countries that are experiencing the start of local COVID-19 outbreaks. Singapore is a small island city-state, and nations with other characteristics might need to adapt and augment Singapore’s approaches to achieve the same level of effectiveness.

**Summary**

**What is already known about this topic?**

First detected in China in late 2019, coronavirus disease 2019 (COVID-19) transmission has spread globally.

**What is added by this report?**

Singapore implemented a multipronged surveillance and containment strategy that contributed to enhanced case ascertainment and slowing of the outbreak. Based on review of the first 100 cases, the mean interval from symptom onset to isolation was 5.6 days and declined after approximately 1 month.

**What are the implications for public health practice?**

A multipronged surveillance strategy could lead to enhanced case detection and reduced transmission of highly infectious diseases such as COVID-19.

## Footnotes

* These authors contributed equally.
